# Source, Distribution, and Risk Estimation of Hazardous Elements in Farmland Soils in a Typical Alluvial–Lacustrine Transition Basin, Hunan Province

**DOI:** 10.3390/ijerph191710971

**Published:** 2022-09-02

**Authors:** Zihan Chen, Bingguo Wang, Chongwen Shi, Yonghui Ding, Tianqi Liu, Junshuai Zhang

**Affiliations:** 1 School of Environmental Studies, China University of Geosciences (Wuhan), Wuhan 430074, China; 2 State Key Laboratory of Biogeology and Environmental Geology, China University of Geosciences (Wuhan), Wuhan 430074, China; 3 Guangzhou Metro Design & Research Institute Co., Ltd., Guangzhou 510010, China

**Keywords:** heavy metals, distribution, source, bioavailable, risk, farmland soil

## Abstract

Increased concentrations of heavy metals in soil due to anthropogenic activities pose a considerable threat to human health and require constant attention. This study investigates the spatial distribution of heavy metals (Cd, Pb, Zn, Sb) and metalloids (As) in a typical alluvial–lacustrine transition basin and calculates the bioavailable forms of elements posing a direct threat. Qualitative and quantitative methods were used to identify the sources of contaminants, after which an ecological risk assessment was conducted. Total (T) As, Pb, and Zn decreased with the depth, whereas Cd and Sb increased in surface (0–20 cm) soil. Bioavailable (Bio) Cd and Pb in the topsoil were regulated by pH and organic matter, whereas Bio-Zn was regulated by soil pH. Within deeper soil layers, the combined effects of pH, organic matter, and clay contents regulated the bio-elements. The results of multiple methods and local investigation showed that TSb (65.3%) was mainly derived from mining activities, TCd (53.2%) and TZn (53.7%) were derived from direct pollution by industrial production and agricultural fertilizers, respectively, and TA (55.6%) was mainly derived from the soil parent material. TPb was related to vehicle exhaust emissions and atmospheric deposition from industrial activities. Although the potential ecological risk in the study area remains relatively low, there is a need for continuous monitoring of the potential ecological risks of Cd and Sb. This study can act as a reference for the prevention and mitigation of heavy metal contamination of alluvial–lacustrine transition basins.

## 1. Introduction

While soil plays a key role in supporting ecosystems and human development, rapid industrialization and urbanization over the last century has resulted in increasingly serious soil pollution [1,2]. The soil pollution survey for 2005–2013 revealed that soil contamination is also a problem in China [3], with 16.1% of samples exceeding the national environmental quality standards. Heavy metals and metalloids are the main soil pollutants, accounting for 82.4% of total pollutants [4]. Therefore, there is an urgent need to identify the distribution and sources of heavy metals in contaminated soils and to evaluate potential ecological risks.

There have been many studies on the diffusion of heavy metals and specific polluted sites [5,6,7,8,9]. While rivers and lakes act as sinks for heavy metals, water resources can also act as heavy metal sources. This is because heavy metals migrate with runoff and settle in agricultural soil through groundwater extraction for irrigation. Throughout human history, settlements have developed near water. The area between the alluvial plain and the lacustrine plain has a flat topology. Therefore, once the river reaches this area, the flow rate decreases, resulting in the deposition of organic matter. This process of deposition increases the fertility of the soil, making it suitable for rice cultivation. Unfortunately, the deposition process also increases the ecological risk of heavy metal and metalloid contamination in this area [10]. However, heavy metal and metalloid pollution usually only receives attention once serious regionalized health challenges are exposed. Studies on the deposition and enrichment of heavy metals at alluvial plain–lacustrine plain transition landscapes remain limited. The Dongting Lake is in north Hunan Province in the middle reaches of the Yangtze River and on the south bank of the Jing River. The lake area is an important grain, cotton, oil, and fisheries production base in Hunan Province. Liu et al. showed that levels of pollution in the Dongting Lake area exceed the background value [11]. The soil of Dongting Lake and various sub-lakes has experienced contamination over the last few decades by multiple heavy metals and metalloids, including As, Cd, Pb, and Zn. Application of the geo-accumulation index revealed the following pollution status: South Dongting Lake (heavy pollution) > East Dongting Lake (severe pollution) > West Dongting Lake (moderate pollution) [12]. Besides being present in irrigation water, heavy metals and metalloids are also widely found as trace elements in the Earth’s crust and soil, which contributes to natural heavy metal background values. Anthropogenic activities have resulted in the accumulation of some heavy metals and metalloids in soil [13]. The anthropogenic activities that contribute to the input of heavy metals and metalloids into soil mainly include industrial production, vehicle exhaust emissions, and agricultural fertilizer emissions [14]. Industrial wastewater is the main source of heavy metals in China. The results of the second National Pollution Source Census in 2017 indicated that the total discharge of heavy metals and metalloids contributing to water pollution in China was 176.40 tons, accounting for 96.6% of total emissions. Key provinces involved in the discharge of heavy metals and metalloids include Guangdong, Zhejiang, Fujian, Hunan, and Guangxi. Therefore, there is an urgent need to characterize the distributions and sources of heavy metals and metalloids in agricultural fields near typical water sources in Hunan Province.

Heavy metal soil contaminants are characterized by high toxicity, bioaccumulation, and persistence. Long-term soil heavy metal enrichment can lead to a decline in soil buffer capacity and fertility, as well as contamination of crop root growth, thereby reducing crop productivity [15]. The accumulation of heavy metals and metalloids can have a serious impact on human health, with health effects related to chronic toxicity, such as skin irritation, immune system damage, and cardiovascular disease [16]. Heavy metals and metalloids present huge potential threats to natural ecosystems and public health. The current methods for studying heavy metals and metalloids include the calculation of contaminant loads, characterizing accumulation, spatial distribution characteristics, pollution, and risk assessment [17]. In recent years, heavy metal source analysis has been widely used in research and practice, and the methods used can be broadly divided into pollution source identification and quantitative analysis approaches. Pollution source identification often uses geographic information systems (GISs), correlation analysis (SPSS), and principal component analysis (PCA) to determine the category of pollution sources. Quantitative analysis of pollution sources uses receptor models, such as chemical mass balance (CMB), positive matrix decomposition (PMF), edge analysis (UNMIX), and geographic detector models (GDMs), to quantitatively determine the contributions of different pollution sources [18]. There is an urgent need to develop an approach for accurate qualitative and quantitative source analysis within ecological planning by combining the above analysis tools. Furthermore, the mobility, bioavailability, and ecotoxicity of heavy metals and metalloids depend largely on their specific chemical forms rather than their total concentrations. Heavy metals and metalloids in water-soluble and ion-exchangeable fractions can be directly absorbed and utilized by organisms. These properties of heavy metals and metalloids have the most direct impact on organisms and are the main driver of phytotoxic effects [19]. Therefore, there is an urgent need to study the water-soluble and ion-exchange fractions of heavy metals. The Zishui River is one of four rivers in the Dongting Lake basin, Hunan Province. The river has a length of 653 km and flows into the South Dongting Lake in Yiyang City. The Zishui River is the main water source for industry, domestic use, and irrigation in Hunan Province. However, the area through which the river flows is rich in mineral resources. Therefore, this region is important for mineral mining and smelting in Hunan Province [20]. The study of the sources, distribution, and ecological risks posed by heavy metals and metalloids is made more difficult by the complex sources of contaminants. The present study has selected farmland soil downstream of Zishui River in a typical alluvial–lacustrine transition basin in the southern Dongting Lake basin. Different forms of the multiple heavy metals and metalloids (As, Cd, Pb, Zn, and Sb) were measured in the soil samples. The spatial distribution of heavy metals and metalloids in the top and profile soil, as well as their drivers, was revealed, and sources of pollution were identified by combining qualitative and quantitative analysis methods. The risks posed by soil heavy metals and metalloids were then assessed. The results of the present study can act as a reference for heavy metal and metalloid migration and transformation, as well as for their biological effects. The results can also act as a scientific reference for the prevention and control of soil heavy metal pollution in the study area.

## 2. Materials and Methods

### 2.1. Study Profile

**Figure 1:**
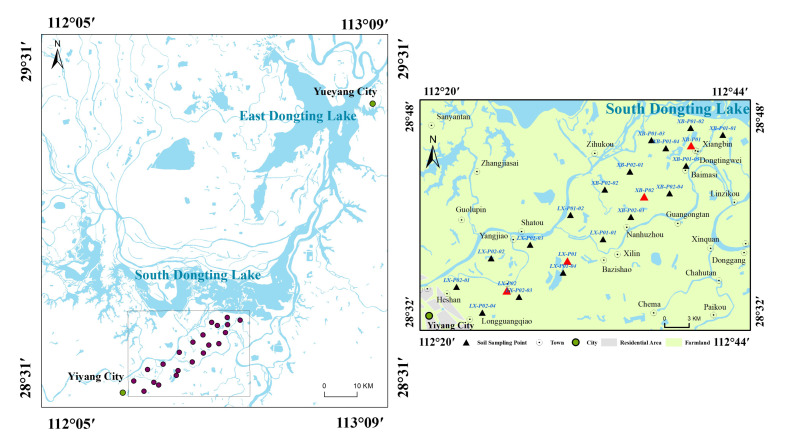
The study area of the present study near South Dongting Lake and the distribution of sampling sites (red triangles represent profile sampling points, whereas black triangles represent topsoil sampling points).

The study area is at the entrance of South Dongting Lake, at the junction of the cities of Yiyang and Yueyang (Figure 1). Administrative districts falling in the study area include the towns of Shatou and Bazishao in Yiyang City and the towns of Nanhuzhou and Xiangbin in Yueyang City. The study area spans E 112°20′ to E 112°44′ Lat. and N 28°32′ to N 28°48′ Lon. and encompasses the Zishui Basin from Yiyang City to South Dongting Lake. The study area falls into a subtropical monsoon climate zone with an annual precipitation of 1100–1800 mm. The terrain of the study area is dominated by flat plains with fertile soil and a dense network of rivers, and the main land use is cultivated land.

Soil samples in the study area from the tillage layer and profile were collected by sampling along the Zishui River from Yiyang City toward Dongting Lake. A global positioning system (GPS) was used to determine sampling location information (Table 1). As shown in Table 1, 17 sampling points were selected for tillage layer soil, and the samples were taken from a depth of 0–30 cm. Investigation of the profile soil involved 4 sampling points at a depth of 160 cm or to the groundwater level (140 cm). Samples at a depth of 0–100 cm were taken in 10 cm intervals, whereas samples at a depth exceeding 100 cm were taken at 20 cm intervals. Each sample consisted of a soil mass of ~1 kg, collected using the quartering method, which was plastic-sealed and bagged for further processing. All samples were air-dried at room temperature, ground, passed through a 0.25 mm nylon sieve, and stored in sealed polyethylene bags until further chemical analysis.

### 2.2. Sample Test Methods

Soil particle size was assessed using a laser particle size analyzer (LS13-320), during which soil samples were pretreated with H_2_O_2_-HCl, purified water, and sodium hexametaphosphate. Soil contents of As, Cd, Pb, Zn, and Sb were extracted in a sequence, during which the water-soluble, ion-exchangeable elements were extracted in sequence. Soil pH was measured using 1:10 carbon dioxide-free water and the ion-selective electrode method (ISE). Soil organic matter content was determined by potassium dichromate redox capacity (VOL). Cd, Pb, and Zn were determined by inductively coupled plasma spectroscopy (ICP-AES). As and Sb were measured by the atomic fluorescence method (AFS). The contents of soil mineral elements (SiO_2_, Al_2_O_3_, TFe_2_O_3_, K_2_O, Na_2_O, CaO, and MgO) were measured by powder X-ray fluorescence spectrometry (XRF). Table 2 provides more information on the extraction methods. The quality monitoring of the sample analysis and testing passed the standard of the “Technical Requirements for Eco-geochemical Evaluation Sample Analysis (Trial)” (DD 2005-03) of the China Geological Survey, and the data quality was considered reliable.

### 2.3. Statistical Analysis of the Data

#### 2.3.1. Pollution Index

The single factor pollution index (PI) and the Nemero comprehensive pollution index (NIPI) are commonly used to evaluate the pollution level of each soil sample:

(1) $$\mathrm{PI}=\frac{C_{i}}{B_{i}}$$

(2) $$\mathrm{NIPI}=\sqrt{\frac{{\mathrm{PI}}_{\max}{}^{2}+{\mathrm{PI}}_{\mathrm{ave}}{}^{2}}{2}}$$

where C_i_ is the measured concentration of element i in the sample and B_i_ is the geochemical background concentration of element i in the Dongting Lake area. PI_max_ is the maximum value of each pollution index in the sample, and PI_ave_ is the average value. According to the pollution index (PI) and the Nemerow comprehensive pollution index (NIPI), heavy metal pollution can be divided into five categories: <0.7, 0.7–1, 1–2, 2–3, and ≥3, which represent no pollution, critical threshold, and low, moderate, and heavy pollution, respectively.

#### 2.3.2. Geo-Accumulation Index

The geo-accumulation index (I_geo_) was proposed by German scientist Muller (1979) [21] to assess contamination levels of bottom sediments and has seen wider use recently in soil pollution assessment. This method not only considers the influence of background values resulting from natural geological processes but also accounts for anthropogenic activities on heavy metal pollution [22]. The formula is as follows:

(3) $$I_{\mathrm{geo}}=\log_{2}\frac{C_{i}}{1.5B_{i}}$$

where C_i_ is the measured concentration of element i in soil and B_i_ is the geochemical background concentration of element i in the Dongting Lake area [23]. The I_geo_ values were classified into seven categories according to I_geo_ values: <0, 0–1, 1–2, 2–3, 3–4, 4–5, and ≥5, which represent uncontaminated, uncontaminated to moderately contaminated, moderately contaminated, moderately to heavily contaminated, heavily contaminated, heavily to extremely contaminated, and extremely contaminated categories, respectively.

#### 2.3.3. Potential Ecological Risk Index

The potential ecological risk index (RI) considers four influencing factors: the concentration of heavy metals and metalloids in the soil, the contaminant type, the toxicity, and the sensitivity of the medium to heavy metal contamination. The method was proposed by Hakanson [24], and the equation is as follows.

(4) $${\mathrm{EI}}_{i}=T_{i}\frac{C_{i}}{B_{i}}$$

(5) $$\mathrm{RI}=\sum_{i=1}^{n}{\mathrm{EI}}_{i}$$

where C_i_ is the measured concentration of element i in the soil, B_i_ is the geochemical background concentration of element i in the Dongting Lake area, T_i_ is the toxicity response factor, EI_i_ is the single potential ecological risk index, and RI is the potential ecological risk of overall pollution. In this study, the toxicity response factors of As, Cd, Pb, and Zn were 10, 30, 5, and 1, respectively. The EI_i_ indices are divided into five categories: <40, 40–80, 80–160, 160–320, and ≥320, and represent low, moderate, considerable, very high, and hazardous categories, respectively. The RI indices are divided into four categories: <150, 150–300, 300–600, and ≥600, and represent low, moderate, considerable, and very high categories, respectively. Since there is no toxicity response for Sb, the present study did not consider the potential ecological risk index of Sb.

#### 2.3.4. Positive Matrix Factorization Model

The main sources of heavy metal emissions from soil were resolved by EPA-PMF (version 5.0), a classical model used to assess quantitative source assignments of heavy metals and metalloids, an improved factor decomposition method developed by Paatero and Tapper nearly 30 years ago [25]. It works by classifying the dataset into several matrices through the following equations.

(6) $$X_{\mathrm{ij}}=\sum_{k=1}^{p}\left(g_{\mathrm{ik}}f_{\mathrm{kj}}{+e}_{\mathrm{ij}}\right)$$

where X_ij_ is the measurement matrix of heavy metal element j in the ith sample, g_ik_ is the contribution matrix of factor k in the ith sample, f_kj_ is the source profile of element j in k factors, and e_ij_ is the residual value of element j in i samples. By simplifying the objective function Q of the model [26], g_ik_ and f_kj_ are determined and expressed using the following equations.

(7) $$Q=\sum_{i=1}^{n}\sum_{j=1}^{m}\left(\frac{x_{\mathrm{ij}}-\sum_{k=1}^{p}g_{\mathrm{ik}}f_{\mathrm{kj}}}{u_{\mathrm{ij}}}\right)=\sum_{i=1}^{n}\sum_{j=1}^{m}{\left(\frac{e_{\mathrm{ij}}}{u_{\mathrm{ij}}}\right)}^{2}$$

If the heavy metal concentration exceeds the MDL, the uncertainty is calculated as shown below, where u_ij_ is the uncertainty of metal j on the ith sample, calculated from the detection limit (MDL) of the element-specific method using the following equation.

(8) $$u_{\mathrm{ij}}=\frac{5}{6}\;\times \;\mathrm{MDL}\;\leq \mathrm{MDL}$$

(9) $$u_{\mathrm{ij}}=\sqrt{{\left(\mathrm{Error}\;\mathrm{Fraction}\;\times \;\mathrm{concentration}\right)}^{2}+{\left(0.5\;\times \;\mathrm{MDL}\right)}^{2}}\geq \mathrm{MDL}$$

The concentration data of the five heavy metals and metalloids in the topsoil and the related uncertainty data were input into PMF5.0, and the factor numbers were set to 2, 3, and 4 in the independent model operation with 20 runs. Q was minimized and stabilized at four factors.

### 2.4. Data Processing

Spatial distributions of total and bioavailable levels of heavy metals and metalloids were mapped using the ArcGIS 10.3((ESRI, Redlands, CA, USA)) and Origin 2018 software (Northampton, MA, USA). SPSS 19.0 software (SPSS Inc., Chicago, IL, USA) was used for statistical and correlation analyses of the bioavailable heavy metal soil levels and soil properties. The heavy metal content in the profile soil was mapped using Origin 2018 software. A positive matrix decomposition model (PMF 5.0) evaluated the heavy metal sources.

## 3. Results and Discussion

### 3.1. Descriptive Statistics of Heavy Metals and Metalloids in Soils

Table 3 shows the heavy metal and metalloid contents of the top and profile soil samples in the study area. The average contents of heavy metals and metalloids in the top and profile soil samples were less than the pollution risk screening values (Sb is not currently included in the soil environmental quality standard) but exceeded the background values of the Dongting Lake area. In particular, the contents of As, Cd, and Sb of topsoil samples exceeded those of background values of the Dongting Lake area by factors of 1.3, 1.2, and 5.6, whereas the As and Sb contents of profile soil samples exceeded the background values by factors of 1.6 and 2.0, respectively. The topsoil contents of Cd, Pb, and Sb generally exceeded those in the profile soil, and elements originating from exogenous pollution were usually concentrated in the surface soil, indicating anthropogenic impacts on Cd, Pb, and Sb. There was no significant difference between As and Zn contents in the surface and profile soils. This result can be attributed to two possible reasons: (1) the elements were mainly derived from the weathering of soil parent material, with less impact by anthropogenic factors; (2) the accumulation of toxic elements in the deep soil due to long-term pollution and long-term leaching in the study area [27]. The present study identified the specific sources of heavy metals through the analysis of spatial distribution characteristics and the application of source identification models.

The order of heavy metals and metalloids in topsoil samples according to overall variability was: Sb (143.72%) > Cd (48.09%) > As (26.06%) > Zn (20.82%) > Pb (10.67%), whereas in profile soil samples it was: Sb (101.50%) > Cd (59.41%) > As (37.63%) > Zn (29.23%) > Pb (18.73%). The variability in Sb in the topsoil and profile soil samples exceeded 100%, indicating that Sb may be strongly influenced by anthropogenic activities in the study area [29].

### 3.2. Spatial Distribution of Heavy Metals and Metalloids in Topsoil

#### 3.2.1. Distribution of Total Heavy Metals and Metalloids

**Figure 2:**
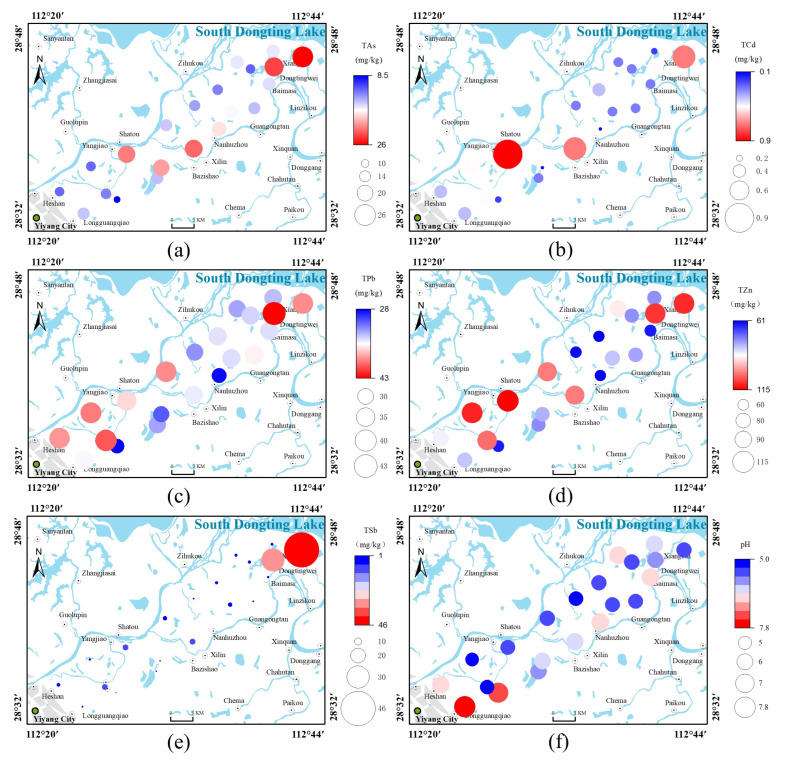
Distributions of the five heavy metals/metalloids and soil pH in the study area: (**a**) As, (**b**) Cd, (**c**) Pb, (**d**) Zn, (**e**) Sb, and (**f**) pH.

Figure 2a–e show the spatial distribution of total heavy metals and metalloids in topsoil. Upstream areas with high concentrations of TAs were mainly distributed in the alluvial sediments near Yangjiao Township, whereas downstream areas included lacustrine sediments far from the riverbank. Areas with high concentrations of TPb were mainly distributed in the lacustrine sediments downstream. No significant difference in the contents of As and Pb at each sampling point was identified, and the content of As and Pb near the town did not increase significantly, indicating that anthropogenic factors had little effect on the soil contents of As and Pb. There was no obvious relationship between the spatial distribution of Pb and known mining activities. Therefore, the origin of As and Pb in soil could be attributed to the weathering and leaching of parent material [30]. Soil TCd and TZn showed similar spatial distributions, with areas of high concentration mainly distributed in the lacustrine sediments near Xiangbin Town and Dongtingwei Town downstream and in alluvial sediments near Yangjiao Township and Shatou Town upstream. This result indicates that high TCd and TZn contents in the topsoil could originate from pollutant emissions from urban dwellings, industrial production, and vehicle exhaust [28]. In particular, high concentrations of TCd can be related to atmospheric deposition and agricultural irrigation [31]. TSb showed considerable spatial variability in the study area, with the highest concentrations (46.38 mg·kg^−1^) distributed in the lacustrine sediments, far from the riverbank downstream of the study area. The area around Yiyang, Hunan Province, is rich in mineral resources and has a high concentration of antimony ore and the largest proven and retained reserves in China. This area contains various large and small antimony ore deposits and mining sites [32]. Therefore, the high concentrations of TSb could be related to mining activities [31]. Although the distributions of the multiple metals in the study area provide some indication as to the sources, the contributions of different forms of metals and metalloids remain unclear.

#### 3.2.2. Distributions of Bioavailable Heavy Metals and Metalloids

The environmental impact of the soil metals and metalloids depends not only on their total concentrations but also on their chemical forms [33]. The seven forms of heavy metals and metalloids, based on their chemical stability in the soil, were extracted using different methods. Additionally, the forms of metals and metalloids were divided according to bioavailability into three fractions: (1) bioavailable, (2) potentially bioavailable, and (3) non-available [34]. The bioavailable fraction represents the sum of the water-soluble and ion-exchangeable fractions, which is characterized by strong activity, ability to migrate, and strong bioaccumulation ability [35].

Table 4 provides an overall summary of the bioavailability of metals and metalloids in the topsoil samples. Cd showed a high bioavailable content, accounting for 37.76% of the total Cd content. The average pH of the soil samples at the observation points in the study area was 6.03, indicating neutral-to-acidic soil in the study area. The pH status of the soil may be a major driver of higher bioavailability of soil Cd in the study area compared to that of other elements. The higher bioavailability of Cd results in the increased potential migration and adsorption of Cd in the study area [36]. In contrast, although there were high total concentrations of As, Pb, Zn, and Sb, their bioavailable components accounted for <3% of total concentrations, indicating a relatively low bioavailability of these metals in the study area [37].

**Figure 3:**
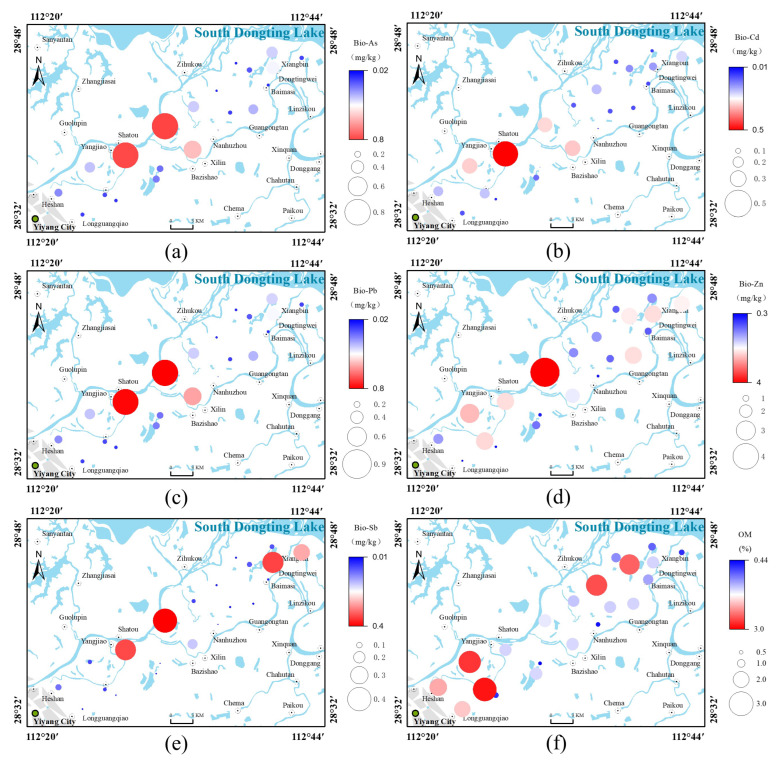
Distributions of bioavailable heavy metals/metalloidsand soil organic matter in the study area: (**a**) As, (**b**) Cd, (**c**) Pb, (**d**) Zn, (**e**) Sb, and (**f**) organic matter.

Figure 3a–e show the spatial distributions of the bioavailable contents of heavy metals and metalloids in topsoil samples. In general, there was clear spatial variation in the distribution of bioavailable metal contents, with concentrations of bioavailable forms of heavy metals and metalloids decreasing from upstream to downstream. Bioavailable forms of As and Pb showed similar spatial distributions, which indicated their origin to be multiple point sources related to both natural weathering and anthropogenic activities. Areas of high concentrations of bioavailable Cd and Zn were mainly concentrated in the central area upstream, whereas areas of low concentrations of bioavailable Cd and Zn showed a scattered distribution downstream. Areas of high concentrations of bioavailable Sb were distributed in the towns of Xiangbin and Dongtingwei downstream and in the towns of Shatou and Yangjiao upstream. This result indicated that high concentrations of Sb were not only related to mining activities but also to industrial production within urban areas. The higher total and bioavailable forms of Sb indicate that further management efforts need to be focused on this heavy metal. In addition, the migration of heavy metals and metalloids by leaching may lead to an underestimation of the ecological risks. Therefore, there is a need to select representative points to explore the distribution of heavy metals and metalloids in deep soils that may be absorbed by soil roots.

### 3.3. Distribution of Heavy Metals and Metalloids in Profile Soil

#### 3.3.1. Distribution of Total Heavy Metals and Metalloids

**Figure 4:**
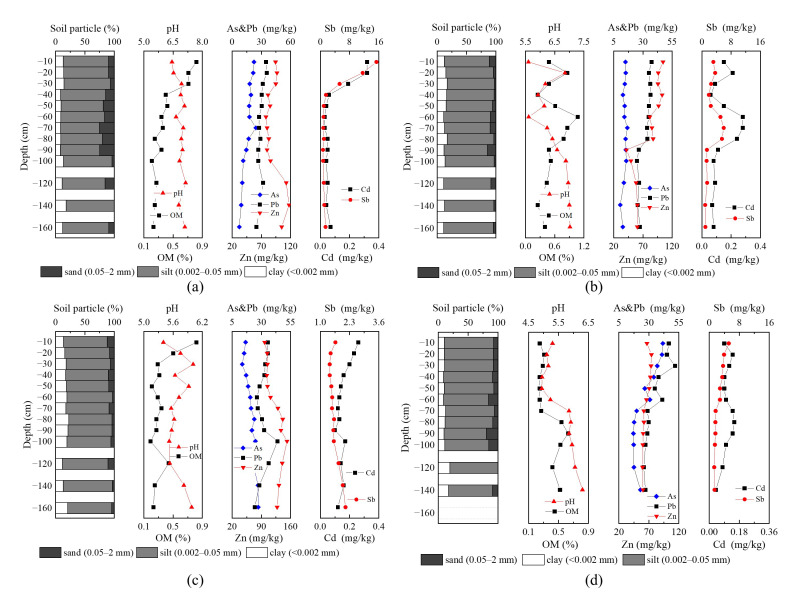
Distribution of heavy metals and metalloids in typical soil profiles: The first column is the vertical distribution of soil particles, the second column is the vertical distribution of pH and organic matter, the third column is the vertical distribution of As, Pb, and Zn, and the fourth column is the vertical distribution of Cd and Sb. (**a**) XBP01, located downstream of Zishui. (**b**) XB-P02, located in the middle and lower reaches of Zishui. (**c**) LX-P01, located in the middle and upper reaches of Zishui. (**d**) LX-P02, located upstream of Zishui.

Figure 4 shows the distributions of total metals and metalloids in four typical profiles. The highest average concentrations of As, Pb, and Zn occurred in LX-P02, upstream of Zishui, which exceeded the background value for the Dongting Lake area by factors of 0.76, 1.12, and 2.18, respectively. The highest concentration of Cd occurred in LX-P01 at 0.16 mg·kg^−1^, exceeding the background value by a factor of 0.51. The highest concentration of Sb occurred in XB-P01, downstream of Zishui, which exceeded the background value by a factor of 2.47. The Sb concentration in XB-P01 was significantly higher than those in other profile soil samples, consistent with the distribution of Sb in topsoil. The five heavy metals and metalloids showed different downward migration characteristics. Cd and Sb showed the highest downward migration at a soil depth of 0–20 cm in XB-P01, LX-P01, and LX-P02 and showed clear surface enrichment characteristics, whereas the concentrations of As, Pb, and Zn all decreased with depth. These results indicated that Cd and Sb mainly originated from exogenous inputs, including atmospheric deposition, mining, and other anthropogenic activities at the soil surface. Higher concentrations of Cd and Sb occurred at a soil depth of 60–80 cm in XB-P02, indicating that leaching resulted in deeper migration of Cd and Sb at this site. A previous study found higher concentrations of metals on the surface, which generally decreased with soil depth, and that the vertical distributions of metals were regulated by soil parent material [38]. Cd and Sb were positively correlated with organic matter content and negatively correlated with pH in the study area. The concentrations of As, Pb, Zn, and clay grains showed consistent trends with soil depth, consistent with the results of Liu et al. [17]. 

#### 3.3.2. Distribution of Bioavailable Metals in Profile Soil

**Figure 5:**
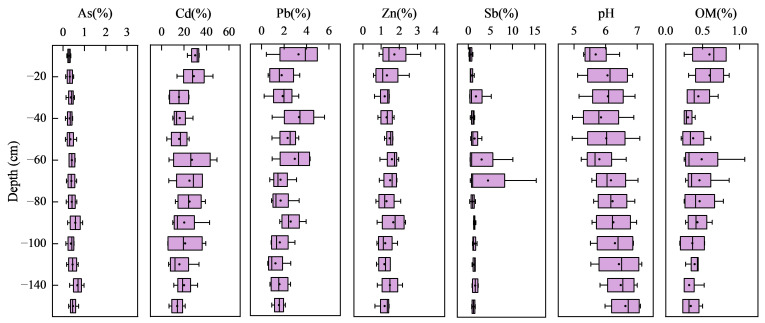
Distribution of the bioavailable fractions of heavy metals and metalloids in the profile soil. (The whiskers represent the minimum and maximum values, respectively, the dots are the mean values, and the three vertical lines in the boxes from left to right represent the 25th percentile, median, and 75th percentile, respectively).

Table 5 shows a summary of the bioavailability of heavy metals and metalloids in the profile soil samples. The metals and metalloids could be ranked according to their bioavailable fractions as: Cd (21.00%) > Pb (2.12%) > Sb (1.58%) > Zn (1.40%) > As (0.41%). The highest bioavailable (water-soluble and ion-exchangeable) portion of Cd could be attributed to its stronger biological mobility and activity compared to other metals, translating into higher potential risks to the ecosystem [39]. Although the bioavailable fractions of As, Pb, Zn, and Sb only accounted for <3% of their respective totals, the ecological risks posed by these bioavailable forms should not be ignored. There were no major changes in the proportions of bioavailable contents of As, Cd, Pb, and Zn with increasing soil depth, with a gradually decreasing trend overall (Figure 5). There was an increased concentration of bioavailable Sb at a depth of 60–80 cm. Sb also showed high variability in the soil profile. These results imply that large quantities of Sb accumulate at the soil surface due to mining activities, resulting in an extremely uneven distribution of this metal in the soil profile.

### 3.4. pH and Organic Matter Are the Main Factors Affecting the Spatial Distribution of Heavy Metals in Soil

**Figure 6:**
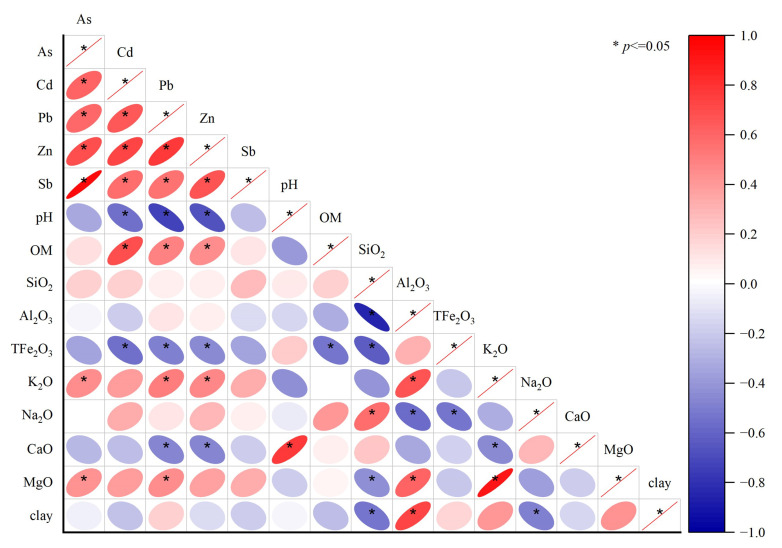
Correlation between bioavailable metals and physicochemical properties in the topsoil of the study area.

**Figure 7:**
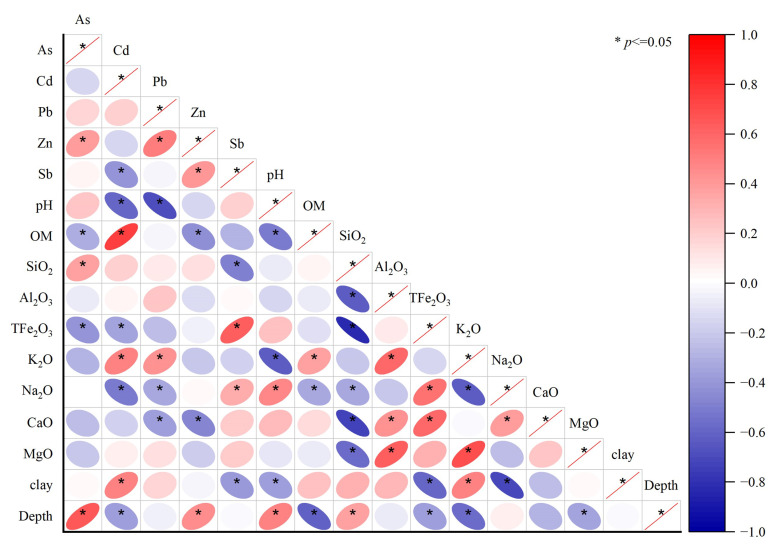
Correlation between bioavailable metals and physicochemical properties in profile soil samples in the study area.

The lowest contents of bioavailable As, Cd, Pb, Zn, and Sb were upstream of the study area, away from the riverbank (Figure 3a–e), showing an opposite pattern to the spatial distribution of pH. The highest pH was far from the riverbank (Figure 2f), and the highest organic matter content was in the upstream and downstream areas near the riverbank (Figure 3f). Bioavailable Cd, Pb, and Zn in soil profile increased with increasing organic matter content and decreased with increasing pH (Figure 5). The present study further clarified the mechanism under which the spatial distributions of bioavailable hazardous elements in soil in the study area were formed. The relationship between bioavailable heavy metals and physicochemical properties in soil was visualized through correlation analysis based on the theory of soil heavy metal behavior (Figure 6 and Figure 7).

For example, bioavailable Cd in paddy soil generally exists in the form of MgCl_2_-Cd and OAC-Cd. An increase in pH results in the hydrolysis of Cd^2+^ to form precipitated Cd(OH)_2_ and Cd_3_(PO_4_)_2_, thereby reducing bioavailable Cd [40]. This law is also supported by the results of Ali et al. [41]. Bioavailable concentrations of Cd and Pb were significantly and positively correlated with organic matter content. This result could be attributed to the chelate function of organic matter in paddy soils, thereby greatly enhancing the bioavailability of Cd [42]. In addition, bioavailable Pb was strongly correlated with the mineral element content of the soil. This could be attributed to the migration of lead(II) on the surface being regulated by the soil mineral adsorption process (such as iron oxide and low-crystalline aluminosilicate). Among soil minerals, metal oxides and low-crystalline aluminosilicates with high surface area and variable surface charge dominated the adsorption of trace metals. The predictive model for Pb(II) adsorption on soil minerals [43] indicated that this process was influenced by the composition of parent materials. There were no obvious correlations between bioavailable Sb and pH, organic matter, and mineral element content, further implying that bioavailable Sb originates from exogenous input and its regulation by anthropogenic activities.

Bioavailable Cd and Pb in profile soil were significantly and negatively correlated with pH, similar to the relationship observed in topsoil. Bioavailable Cd also showed a significant positive correlation with clay content. Soil with high clay content is also high in dissolved organic matter, which may facilitate the adsorption of heavy metals and metalloids in soil, resulting in increased concentrations of bioavailable elements [44]. However, bioavailable Zn was significantly and negatively correlated with organic matter. There was strong clustering of organic matter and bioavailable Zn in the soil. Zn may form stable metal chelates through complexation and adsorption, thereby reducing bioavailable Zn concentration. Bioavailable Zn concentration increased with increasing soil depth. Although bioavailable Sb was not significantly correlated with pH and organic matter, it was significantly correlated with clay content and the soil concentrations of SiO_2_, Fe_2_O_3_, and Na_2_O. This result further implies that Sb in the topsoil originated from exogenous contamination, which did not extend to the deeper soil layers, whereas bioavailable Sb in deeper soils originated from the soil parent material.

In general, the concentrations of bioavailable metals were closely related to soil pH and organic matter content. Soil pH regulates the physical, chemical, and biological processes impacting metal processes in soil, including dissolution, precipitation of metal solid phases, metal complexation, and acid-base reactions [45]. The decrease in soil pH changes the stable binding state of heavy metals and metalloids and promotes the dissolution of metals, thereby increasing the contents of bioavailable elements in the soil [37]. An increase in pH will increase the negative charge on the soil colloid surface, resulting in the precipitation of carbonates and the formation of hydroxides by bioavailable heavy metals, thereby reducing the mobility of heavy metals [46]. Organic matter in soil can form stable substances that are different from ions containing metals, which, in turn, affects the mobility and morphological composition of soil heavy metals and metalloids [47].

### 3.5. Analysis of the Sources of Heavy Metals and Metalloids 

**Figure 8:**
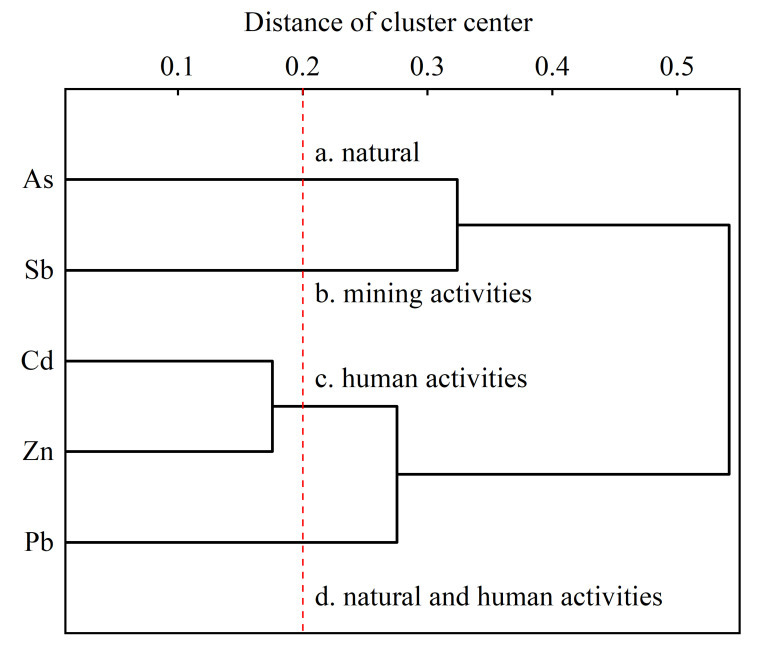
The sources of soil heavy metals and metalloids, indicated by cluster analysis.

The present study conducted a cluster analysis to identify the sources of soil heavy metals and metalloids in the study area. This involved using a tree graph of the average connection between groups and the application of the systematic clustering method using Pearson’s correlation as the metric standard. Data outliers were eliminated, and the data were standardized before clustering analysis. Therefore, the influences of outliers and specific variables on clustering could be ignored. The distance of the cluster center of 0.2 indicated that As, Cd, Pb, Zn, and Sb in the study area could be divided into four categories (Figure 8): (1) As; (2) Sb; (3) Cd and Zn; and (4) Pb. These different categories indicated that the heavy metals and metalloids in the different categories had different sources. This result, combined with that of the spatial distributions of metals in the study area, implies that soil As concentration is mainly regulated by natural factors, Sb is related to mining activities, Cd and Zn are mainly influenced by industrial production and agricultural activities, and Pb is related to both natural weathering and anthropogenic activities.

**Figure 9:**
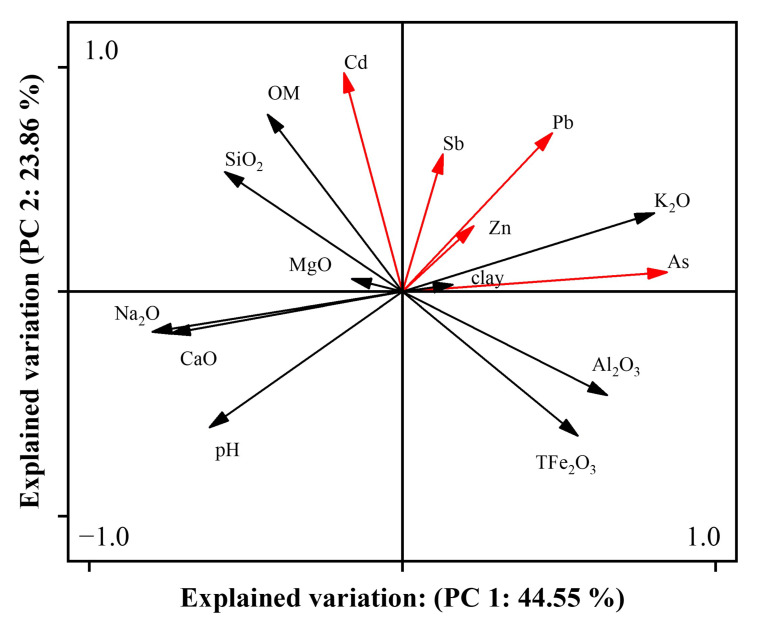
The results of principle component analysis, indicating the sources of soil heavy metals and metalloids. (The red line represents heavy metals and metalloids, the black line represents physical and chemical properties).

As shown in Figure 9, the present study conducted principal component analysis (PCA) on soil heavy metal contents, mineral element contents, and soil physical and chemical properties to further reveal the sources of soil metals and metalloids in the study area. The number of factors generated by PCA represents the total number of possible sources of variation in the chemical data. The extracted first two components, with eigenvalues > 1, explained 68.4% of the total variance in the dataset, with principal component 1 (PC1) and PC2 explaining 44.6% and 23.9% of the total variance, respectively. PC1 showed strong positive loading on As, Pb, K_2_O, Al_2_O_3_, and TFe_2_O_3_. This result indicated that As and Pb combined with Fe in the soil to form insoluble compounds or co-precipitated with Fe, Al, and other hydroxides. The concentrations of As and Pb were close to the study area background values, and their spatial distributions were significantly different from those of other metals. This implies that PC1 represents natural sources of As and Pb. PC2 showed a strong positive load on Cd, Pb, Zn, Sb, mineral element SiO2, and organic matter, indicating that the heavy metal contents were affected by soil clay and organic matter [47]. Soil organic matter is highly capable of retaining or immobilizing metals, thereby affecting their migration and distribution in the soil [48]. The topsoil concentrations of Cd and Sb significantly exceeded those in profile soil samples, and areas of high concentrations of Cd, Zn, and Sb were concentrated in villages and around towns. PC2 is considered to represent anthropogenic sources of soil metals, such as industrial production and agricultural activities. Pb showed strong positive loadings on PC1 and PC2, indicating that Pb was influenced by both natural weathering and anthropogenic activities.

**Figure 10:**
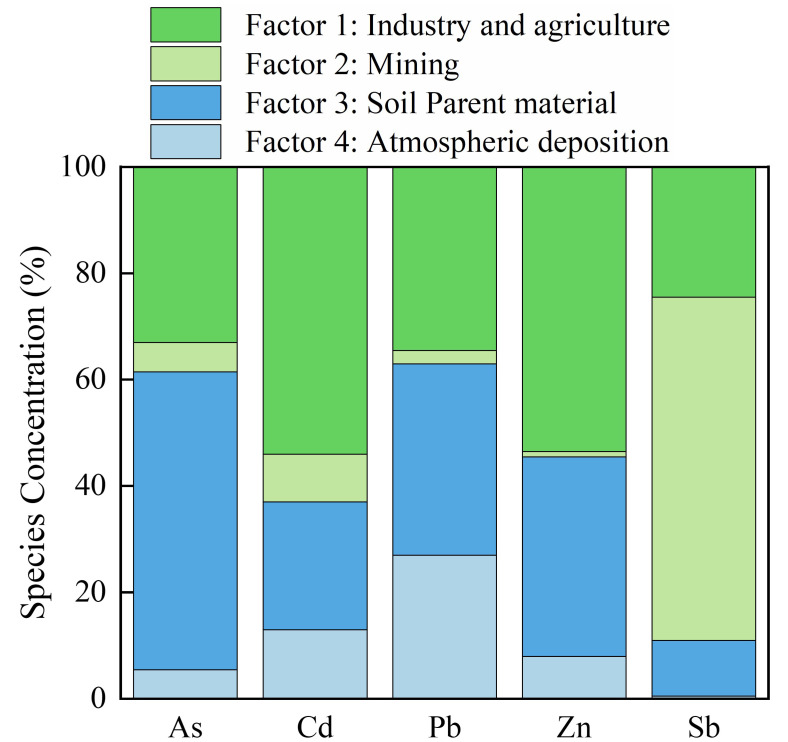
Sources of soil heavy metals and metalloids in the study area according to the results of the positive matrix factorization (PMF) model.

The present study further quantitatively analyzed the sources of heavy metals and metalloids in soil using the PMF model. The PMF model is a source analysis method recommended by the United States Environmental Protection Agency (USEPA). The PMF model has been widely and effectively applied for the quantitative identification of pollutants in air, water, and sediment [49]. The highest correlations between the PMF-predicted and measured values were identified for As, Pb, Zn, and Sb (r^2^ > 0.9), followed by a high correlation for Cd (r^2^ = 0.63). These results indicated that the four factors selected as drivers in the PMF model could be regarded as the sources of heavy metals and metalloids in the study area. Figure 10 shows the contributions to soil heavy metals and metalloids among the different sources, as estimated from the PMF model. Factor 1 was the dominant source of Cd (53.2%) and Zn (53.7%). Cd and Zn showed similar spatial distributions and residential activities in towns, and emissions by industry were the main sources of soil Cd and Zn. Other identified sources of Cd and Zn include livestock manure, fertilizers, pesticides, and the plastic film widely used in agricultural soils [50]. Previous studies have determined that 5.5 × 10^7^ tons of chemical fertilizers are applied to cultivated soil in China every year [51]. The long-term application of chemical fertilizers can lead to the accumulation of heavy metals and metalloids in the soil, thereby resulting in serious environmental challenges. The results of the National Soil Status Survey in 2014 indicated that the heavy metal concentrations at 16.1% of sites in China exceed the national standards, with 7% of sites showing Cd concentrations that exceed the standard. Crop production in the study area is often accompanied by the application of high levels of chemical fertilizer and pesticides. The higher concentrations of Cd and Zn in agricultural fields compared to background values indicate that this factor is associated with emissions of heavy metals and metalloids from industrial production and agricultural fertilization.

Factor 2 was the main factor explaining soil Sb (65.3%). Sb is a dominant element in China and is concentrated in 12 antimony-forming belts [52]. The study area is in Yiyang, Hunan Province, and this area is rich in mineral resources, with the largest concentration of antimony ore deposits in China. Extensive Sb mining in this area has had a considerable impact on the surrounding environment. A study by Mo et al. [53] showed that the average Sb concentration of agricultural soils in the antimony mining areas exceeded that of the Dutch soil standard by a factor of 695. The mining area was seriously affected by Sb pollution. In summary, Factor 2 was defined as being related to mining activities. 

Factor 3 was the main factor explaining As (55.6%) and Pb (36.2%). The contents of both these elements in most soil samples were close to the natural background levels. Soil profile analysis showed that there were no significant differences in the concentrations of As and Pb between topsoil and profile soil samples and that they showed low spatial variability. These results imply that parent material and the process of pedogenesis were the main factors affecting the content and distribution of As and Pb in soil. Thus, Factor 3 was defined as the natural soil parent material.

Factor 4 was the dominant factor explaining Pb (26.3%) and Cd (13.1%). Atmospheric deposition is usually considered to be an important source of Pb and Cd accumulation in soil [54]. Past studies have concluded that vehicle emissions account for about two-thirds of global lead emissions [55]. Pb is released into the atmosphere, after which atmospheric deposition of Pb contaminates surface water and soil [56]. Coal mining and combustion emissions may also be important sources of soil Pb and Cd through atmospheric deposition [57]. Therefore, Factor 4 was defined as being related to atmospheric deposition.

Anthropogenic activities were the main sources of the five elements. Sb (65.3%) was mainly derived from mining activities, Cd (53.2%) and Zn (53.7%) were related to industrial production and application of agricultural fertilizer, and As (55.6%) was mainly derived from the weathering of the soil parent material. Sources of soil Pb included both natural and anthropogenic factors, with the latter including agricultural fertilizer, vehicle emissions, and industrial production.

### 3.6. Risk Assessment of Soil Heavy Metals and Metalloids 

**Figure 11:**
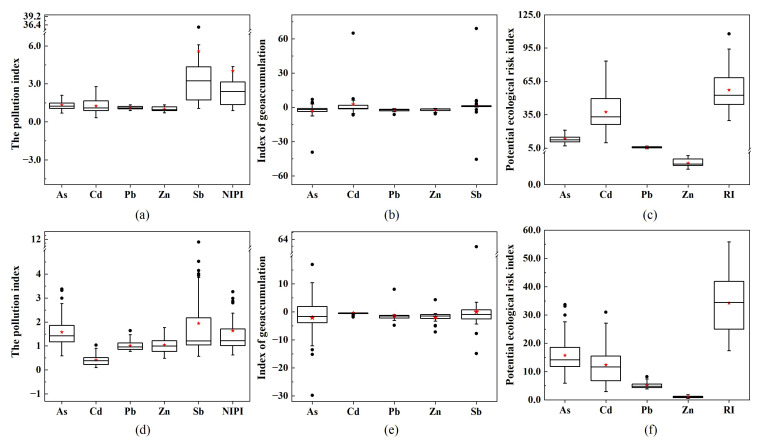
Geological accumulation index, potential ecological risk index, and the single factor index of heavy metals and metalloids for topsoil (**a**–**c**) and profile soil (**d**–**f**). (The circles at the top and bottom of the box plot correspond to the maximum and minimum values, respectively. The pentagrams in the box plot are the mean values. The horizontal lines at the top, middle, and bottom of the box plot represent the 75th percentile, median, and 25th percentile, respectively.)

The present study used relevant indices to evaluate the overall risk of heavy metals in the study area based on an understanding of the biological toxicity of heavy metals. The present study applied several methods to evaluate the risk posed by soil metals and metalloids. Figure 11a,d show the results of the single factor pollution index evaluation. The rank of the different metals according to their pollution levels in the soil of the study area was: Sb > As > Cd > Pb > Zn. The single factor pollution indices of As, Cd, and Pb were less than 2, indicating a low pollution level, whereas that of Zn was less than 1, within the critical threshold. This result indicated that besides Sb, all the studied metals had a low contribution to soil heavy metal pollution. In contrast, 57% of soil samples showed Sb concentrations falling within the heavy pollution level, with a further 9% of the samples showing a moderate pollution level. Therefore, Sb was the main contributor to metal pollution in the study area. The increase in soil Sb concentration was affected by geological and anthropogenic factors. The weathering of parent rock, mining, and industrial processing of Sb-containing ores resulted in soil Sb transitioning from a relatively stable mineral to a mineral that readily undergoes changes to its ionic or granular state. These changes to the state of Sb facilitate its migration into the soil, resulting in increasing environmental Sb concentrations [58]. There are many antimony ore mines in the central Hunan area [59], including the largest antimony ore mine globally, with a mining history of nearly 130 years [60]. The production of Sb through intensive mining activities results in the input of Sb to the soil, rivers, and atmosphere. Sb is enriched in surface soil through irrigation, atmospheric deposition, and other activities, thus becoming a dominant metal contributing to soil heavy metal pollution [61]. The rank of the five heavy metals and metalloids in the profile soil samples according to contamination levels was: Sb > As > Zn > Pb > Cd. The single factor pollution index of Cd was less than 0.7, representing a non-pollution level, whereas the remaining four elements showed low pollution levels in the profile soil samples. The levels of pollution of Sb and Cd in profile soils were significantly lower than those in topsoil, indicating that topsoil Cd and Sb mainly originate from exogenous input, primarily antimony mining and residential activities. The levels of As and Zn pollution in profile soil samples exceeded those in topsoil, indicating that As and Zn may be affected by naturally occurring minerals.

The results of the Nemerow comprehensive pollution index (Figure 11a,d) showed that the average pollution index (NIPI) value for topsoil was 4.03, falling into the category of heavy pollution. The average NIPI for profile soil samples was 1.63, falling in an overall moderate pollution level. The topsoil showed serious heavy metal pollution, and there should be a particular focus on the management of Sb contamination of topsoil.

The geo-accumulation index was calculated using the metal background concentrations for the Dongting Lake basin (Figure 11b,e). The rank of the different studied elements according to the index of topsoil was: Cd > Sb > Zn > Pb >As. The geo-accumulation index values of Pb and Zn in the topsoil were less than zero, indicating that their topsoil concentrations did not exceed the contamination threshold. These results indicate that As and Cd in topsoil originate from mostly natural sources, with a small contribution from point sources. Sb in topsoil exceeded the limit by 14.3%, and most soil samples were classified as lightly contaminated, whereas a few were classified as extremely contaminated, indicating that local antimony mining had a significant impact on soil pollution. However, no limit is currently set for soil Sb in China. There should be further research on the impact of Sb on the ecological environment and human health, and corresponding pollution restriction policies should be established to mitigate damage to the ecological environment and the potential threat to human health resulting from regional heavy metal pollution. In addition, accumulated As, Pb, and Zn in the profile soil samples exceeded those in topsoil.

Figure 11c,f show the calculated potential ecological risk index (ERI) values for the study area. The ERI values of As, Pb, and Zn of the samples were all less than 40, indicating that these elements pose less risk to the ecological environment. Of the topsoil samples, 61.9% showed Cd concentrations representing low potential ecological risk, whereas 4.8% of samples fell into the considerable risk category. The ERI results indicate that all soil samples are of low potential ecological risk if the influence of Sb is ignored. It is worth noting that the potential ecological risk of Cd in topsoil is significantly higher than those of profile soil samples.

## 4. Conclusions

(1) The average concentrations of heavy metals and metalloids in the topsoil and profile soil samples were less than the pollution risk screening values. However, they exceeded the heavy metal background values for the Dongting Lake area. The variability of Sb in the topsoil and profile soil samples exceeded 100%, indicating that soil Sb was strongly regulated by anthropogenic activities in the study area.
(2) The bioavailability of Cd and Pb in topsoil was mainly influenced by soil pH and organic matter, whereas that of topsoil Zn was mainly influenced by pH. Soil pH, organic matter, and clay content had a combined effect on bioavailable Cd in the profile soil. pH was the main factor affecting bioavailable Pb, organic matter was the main regulator of bioavailable As and Zn, and soil clay content was the main factor affecting bioavailable Sb.
(3) Qualitative and quantitative analyses of the sources of soil metals and metalloids revealed that Sb (65.3%) was mainly derived from mining activities, Cd (53.2%) and Zn (53.7%) were related to industrial production and agricultural fertilization emissions, and As (55.6%) was mainly derived from weathering of the soil parent material. Pb in soil was related to both natural and anthropogenic factors, with the latter including agricultural fertilization, vehicle emissions, and the atmospheric deposition of industrial emissions.
(4) There were low soil pollution levels of As, Pb, and Zn in the top and profile soil samples. Heavy metal pollution in topsoil was relatively serious compared to that in the profile soil samples, particularly for Sb; this should receive increasing attention. Although there is a relatively low potential ecological risk in the study area, there is a need for increased attention to the potential ecological risk of Cd in topsoil.

## Figures and Tables

**Table 1:** Basic information for the soil samples.

Sample No.	Longitude /°	Latitude /°	Sample Depth /cm	Sediment Type I	Sediment Type II
XB-P01-01	112.7136	28.7606	0–30	Downstream lacustrine deposits	Offshore
XB-P01-02	112.6733	28.7678	0–30	Downstream lacustrine deposits	Nearshore
XB-P01-03	112.6244	28.7539	0–30	Downstream lacustrine deposits	Nearshore
XB-P01-04	112.6426	28.7453	0–30	Downstream lacustrine deposits	Nearshore
XB-P01-05	112.6680	28.7261	0–30	Downstream lacustrine deposits	Offshore
XB-P02-01	112.5980	28.7189	0–30	Midstream alluvium	Nearshore
XB-P02-02	112.5668	28.6988	0–30	Midstream alluvium	Nearshore
XB-P02-03	112.5999	28.6690	0–30	Midstream alluvium	Offshore
XB-P02-04	112.6479	28.6954	0–30	Midstream alluvium	Offshore
LX-P01-01	112.5654	28.6441	0–30	Midstream alluvium	Offshore
LX-P01-02	112.5179	28.6738	0–30	Midstream alluvium	Nearshore
LX-P01-03	112.4743	28.6369	0–30	Upstream alluvium	Nearshore
LX-P01-04	112.5160	28.6067	0–30	Upstream alluvium	Offshore
LX-P02-01	112.3832	28.5894	0–30	Midstream alluvium	Nearshore
LX-P02-02	112.4259	28.6216	0–30	Upstream alluvium	Nearshore
LX-P02-03	112.4614	28.5794	0–30	Upstream alluvium	Offshore
LX-P02-04	112.4158	28.5616	0–30	Upstream alluvium	Offshore
XB-P01 *	112.6745	28.7479	0–160	Downstream lacustrine deposits	Offshore
XB-P02 *	112.6164	28.6912	0–160	Midstream alluvium	Offshore
LX-P01 *	112.5212	28.6198	0–160	Upstream alluvium	Offshore
LX-P02 *	112.4463	28.5866	0–140	Upstream alluvium	Offshore

* indicates the profile soil. Soil texture analysis classified all soils as silt loam.

**Table 2:** Methods used to process soil samples in the current study.

No.	Forms	Extraction Methods
I	water-soluble fraction	2.5000 g sample 25 mL water extraction
II	ion-exchangeable fraction	The residue was extracted with 25 mL MgCl_2_ solution
T	Total content	0.1000 g soil was digested with 2:2:1 HNO_3_-HF-HClO_4_ at 180–210 °C

**Table 3:** Basic physical and chemical properties of heavy metals and metalloids in soils.

Element		As	Cd	Pb	Zn	Sb	pH	OM
Units		mg·kg^−1^					-	%
Topsoil	Max	25.9	0.86	42.6	114.5	46.38	7.78	2.89
	Min	8.5	0.1	27.6	60.8	1.4	5.03	0.44
	Mean	16.33	0.39	34.74	84.97	7.35	6.03	1.55
	SD	4.26	0.19	3.71	17.69	10.56	0.68	0.69
	CV(%)	26.06	48.09	10.67	20.82	143.72	11.32	44.67
Profile Soil	Max	41.6	0.32	51.8	151.5	15.35	7.14	1.07
	Min	7.3	0.03	24.39	41.7	0.75	4.94	0.19
	Mean	19.46	0.13	31.97	89.22	2.54	6.15	0.43
	SD	7.32	0.07	5.99	26.08	2.58	0.64	0.21
	CV(%)	37.63	59.41	18.73	29.23	101.5	10.43	48.57
Background value of Dongting Lake area [28]		12.35	0.31	31.69	86.1	1.32		
Risk screening value (GB 15618-2018)		45	0.3	80	200		pH ≤ 5.5	
		40	0.4	100	200		5.5 < pH ≤ 6.5	
		35	0.6	140	250		6.5 < pH ≤ 7.5	

**Table 4:** The bioavailable fractions of the heavy metals and metalloids in topsoil.

	As (%)	Cd (%)	Pb (%)	Zn (%)	Sb (%)	pH (-)	OM (%)
Max	6.02	59.96	6.66	5.16	8.72	7.78	2.89
Min	0.16	9.17	0.22	0.39	0.72	5.03	0.44
Mean	1.81	37.76	2.31	2.23	2.11	6.03	1.55
SD	1.38	13.61	1.67	1.10	1.82	0.68	0.69
CV (%)	76.17	36.05	72.48	49.21	86.31	11.32	44.67

**Table 5:** The bioavailable fractions of the heavy metals and metalloids in profile soil samples.

	As (%)	Cd (%)	Pb (%)	Zn (%)	Sb (%)	pH (-)	OM (%)
Max	0.98	49.37	5.59	3.18	15.40	7.14	1.07
Min	0.10	4.55	0.23	0.58	0.10	4.94	0.19
Mean	0.41	21.00	2.12	1.40	1.58	6.14	0.42
SD	0.21	12.12	1.32	0.53	2.45	0.65	0.21
CV (%)	50.86	57.71	62.12	38.15	154.63	10.55	49.76

## Data Availability

Not applicable.
